# Changes in Peripheral Immune Cells after the Third Dose of SARS-CoV-2 mRNA-BNT162b2 Vaccine and Disease Outcomes in Cancer Patients Receiving Immune Checkpoint Inhibitors: A Prospective Analysis of the Vax-on-Third-Profile Study

**DOI:** 10.3390/cancers15143625

**Published:** 2023-07-14

**Authors:** Fabrizio Nelli, Carlo Signorelli, Agnese Fabbri, Diana Giannarelli, Antonella Virtuoso, Julio Rodrigo Giron Berrios, Eleonora Marrucci, Cristina Fiore, Marta Schirripa, Mario Giovanni Chilelli, Francesca Primi, Valentina Panichi, Giuseppe Topini, Maria Assunta Silvestri, Enzo Maria Ruggeri

**Affiliations:** 1 Medical Oncology Unit, Department of Oncology and Hematology, Central Hospital of Belcolle, 01100 Viterbo, Italy; fabrizio.nelli@asl.vt.it (F.N.);; 2 Thoracic Oncology Unit, Department of Oncology and Hematology, Central Hospital of Belcolle, 01100 Viterbo, Italy; 3 Biostatistics Unit, Scientific Directorate, Fondazione Policlinico Universitario A. Gemelli, IRCCS, 00168 Rome, Italy; 4 Cytofluorimetry Unit, Department of Oncology and Hematology, Central Hospital of Belcolle, 01100 Viterbo, Italy; 5 Microbiology and Virology Unit, Department of Oncology and Hematology, Central Hospital of Belcolle, 01100 Viterbo, Italy

**Keywords:** SARS-CoV-2, COVID-19 vaccine, third dose, NK cells, solid tumors, advanced disease, immune checkpoint inhibitors, survival

## Abstract

**Simple Summary:**

International standards recommend booster immunization against SARS-CoV-2 in advanced cancer patients undergoing active treatment. Little is known about the relationship between cell-mediated immune responses after vaccination and disease outcomes on immune checkpoint blockade. In this study, we sought to investigate the effects of the third dose of tozinameran on dynamic changes in absolute peripheral lymphocyte counts and their impact on the survival of patients receiving anti-PD-1/PD-L1 agents. The booster dose induced a significant increase in NK cell counts, which correlated with an improved antibody response and a decreased rate of breakthrough infections. Patients with higher levels of NK cells after the third immunization also had a significantly reduced risk of treatment failure in the following six months and longer overall survival. The results from this study provide evidence that COVID-19 vaccination is unlikely to blunt the clinical efficacy of immune checkpoint inhibitors. Our findings also suggest a favorable interaction mediated by the NK cell response, which is consistent with previous insights and needs further confirmation.

**Abstract:**

Background: Anti-SARS-CoV-2 mRNA vaccines can deeply affect cell-mediated immune responses in immunocompromised recipients, including cancer patients receiving active treatments. The clinical implications of changes in peripheral blood lymphocyte subsets following the third dose of mRNA-BNT162b2 vaccination (tozinameran) in patients on immune checkpoint blockade are not fully understood. We conducted a prospective analysis of the Vax-On-Third-Profile study to evaluate the impact of circulating lymphocyte dynamics on disease outcomes in this subgroup of patients. Methods: Recipients of booster dosing who had received before vaccination at least one course of an anti-PD-1/PD-L1 treatment for an advanced solid tumor were eligible. Immunophenotyping of peripheral blood was performed before the third dose of tozinameran (timepoint-1) and four weeks later (timepoint-2) to quantify the absolute counts of lymphocyte subpopulations, including CD3^+^CD4^+^ T cells, CD3^+^CD8^+^ T cells, B cells, and NK cells. Logistic regression was used to analyze the relationship between lymphocyte subsets and durable clinical benefit (DCB). The log-rank test and Cox regression model were applied to evaluate the relationship between lymphocyte subpopulations and both vaccine-related time-to-treatment failure (V-TTF) and overall survival (OS). Results: We included a total of 56 patients with metastatic disease who were given a third dose of tozinameran between 23 September and 7 October 2021 (median age: 66 years; male: 71%). Most recipients had a diagnosis of lung cancer and were being treated with pembrolizumab or nivolumab. Compared to baseline, the third immunization resulted in an incremental change in the median counts of all lymphocyte subpopulations, which was statistically significant only for NK cells (*p* < 0.001). A significant correlation was found between NK cell counts and DCB at timepoint-2 (*p* < 0.001). Multivariate logistic regression analysis of DCB confirmed the predictive significance of high-level NK cell counts (*p* = 0.020). In multivariate Cox regression analysis, high-level NK cell counts independently predicted longer V-TTF [HR 0.34 (95% CI 0.14–0.80), *p* = 0.014] and OS [HR 0.36 (95% CI 0.15–0.89), *p* = 0.027]. Conclusions: Our data suggest expansion of NK cell counts as the most noteworthy change in circulating lymphocytes after the third dose of tozinameran in cancer patients receiving PD-1/PD-L1-targeted agents. This change correlated with enhanced therapeutic efficacy, improving the rate of disease control, and prolonging survival outcomes. Similar findings have not been previously reported, implying that they have proof-of-concept value and warrant further confirmation.

## 1. Introduction

Despite its recent declaration that COVID-19 no longer constitutes a public health emergency of international concern, the World Health Organization (WHO) recommends maintaining efforts to increase vaccine coverage against SARS-CoV-2 for all people in high-priority groups, including most cancer patients with advanced disease [1]. Patients with solid malignancies undergoing active treatments were able to produce a more intensive antibody response after receiving a third dose of a mRNA-based vaccine than was achieved with the initial two-dose series [2]. Boosting humoral immunity would provide protection against symptomatic COVID-19, even in those whose response has weakened or waned over time [3,4]. Specific T cell reactivation is believed to be the main mechanism underlying the enhancement of humoral immunogenicity [5,6]. Current insights suggest the contribution of an early natural killer (NK) cell activation in the generation of immunity against SARS-CoV-2 following mRNA-based vaccination [7,8]. Although knowledge is still limited, subsequent research has confirmed a growing increase in NK lymphocytes after booster dosing, which also positively correlated with clonal expansion of interferon (IFN) γ-producing CD4^+^ T cells [9]. In the Vax-On-Third-Profile study, we observed a significant incremental variation in absolute counts of C8^+^ T cell and NK cell subsets after the third dose of mRNA-BNT162b2 vaccine (tozinameran), which is consistent with previous findings [10]. Although these dynamic changes in peripheral lymphocytes were not related to increased antibody titers, their evidence raised the hypothesis of a potential interaction with the clinical effects of immune checkpoint inhibitors (ICIs) [11]. Systemic immunity plays an essential role in the success of cancer immunotherapy [12]. Immune cell dynamics can accurately reflect the complex interplay between tumor microenvironment plasticity and the influence of external factors in the context of immune checkpoint blockade [13]. Since it has been suggested that changes in circulating immune cell fractions modulate the activity of agents targeting the PD-1/PD-L1 immunoregulatory pathway, whether booster doses of vaccines can affect the efficacy of these treatments still remains to be clarified [14,15]. We conducted a prospective subgroup analysis of the Vax-On-Third-Profile study to investigate whether dynamic changes in peripheral blood lymphocytes following the third dose of tozinameran have an impact on clinical outcomes of advanced cancer patients receiving anti-PD-1/PD-L1 agents.

## 2. Materials and Methods

### 2.1. Study Design and Participants

The Vax-On-Third-Profile was a prospective, observational investigation, whose design and main outcomes have already been described (clinical study identifier: EudraCT number 2021-002611-54) [10]. The study adhered to the Strengthening the Reporting of Observational Studies in Epidemiology (STROBE) standards. The referring Ethics Committee gave approval for the study, and all participants granted written informed consent (protocol number: 1407/CE Lazio1). Patients were allowed to participate in this subgroup analysis if they had a histological diagnosis of solid tumor that was either locally advanced or metastatic and had received at least one dose of an agent that targets either PD-1 (nivolumab, pembrolizumab, or cemiplimab) or PD-L1 (atezolizumab, durvalumab, or avelumab) before the third dose of tozinameran. Eligible patients were also required to have no evidence of progression when restaged within eight weeks prior to the third dose and at least one subsequent reassessment performed within six months of the third dose. Participants were tested for IgG antibody levels against the SARS-CoV-2 spike protein (RBD-S1) and lymphocyte subpopulation counts. The primary endpoints were clinical benefit and survival outcomes based on peripheral lymphocyte levels. The efficacy of anti-PD-1/PD-L1 therapies was determined by durable clinical benefit (DCB), which refers to any objective response (complete or partial) or stabilization of disease as per the Response Evaluation Criteria in Solid Tumors (RECIST 1.1) that lasts more than six months after booster vaccination. The patients who achieved DCB were compared to those who did not (NCB, no clinical benefit). Survival endpoints included vaccine-related time-to-treatment failure (V-TTF, defined as the length of time between treatment with an anti-PD-1/PD-L1 agent prior to the booster dose and its withdrawal for any reason) and overall survival (OS, meaning the time between the start of anti-PD-1/PD-L1 treatment and death from any cause). Patients who did not experience disease progression or died were censored as of the date of the interim analysis (30 April 2023).

### 2.2. Peripheral Blood Assessments

The collection of blood samples for serological and immunological assays was performed the day before the third dose of tozinameran (timepoint-1) and four weeks afterward (timepoint-2). As directed by the manufacturer, anti-RBD-S1 IgG antibodies were detected using the SARS-CoV-2 IgG II Quant assay on the ARCHITECT i2000sr automated platform (Abbott Laboratories, Diagnostics Division, Sligo, Ireland) [16]. The data were presented as arbitrary units (AU)/mL over a linear range that was expanded to 80,000 AU by an automated dilution. A WHO international standard for anti-SARS-CoV-2 immunoglobulin testing converted serological titers from AU to binding antibody units (BAU) (1 Abbott AU is equivalent to 0.142 WHO BAU) [17]. The BD FACSCanto II system and BD FACSCanto clinical software (BD Biosciences, San Jose, CA, USA) were used in accordance with the producer’s instructions to retrieve peripheral lymphocyte subsets [18]. The panel for staining included the monoclonal antibodies CD3 FITC, CD4 PE-Cy7, CD8 APC-Cy7, CD19 APC, CD45 PerCP-Cy5.5, CD56 PE, and CD16 PE (BD Biosciences, San Jose, CA, USA). The BD Multitest 6-color TBNK reagent was used to calculate the absolute counts of T helper cells (CD3^+^CD4^+^), T cytotoxic cells (CD3^+^CD8^+^), B cells (CD19^+^), and NK cells (CD56^+^CD16^+^). The results were displayed as absolute cell counts/µL for each lymphocyte subset. Supplementary Figure S1 details the operating procedure and gating strategy of flow cytometry assessments.

### 2.3. Statistical Analysis

Normally distributed variables were described using a mean with standard deviation, while skewed variables were described using a median with a 95% confidence interval or interquartile range (IQR). The Mann–Whitney *U* test for continuous variables and the Pearson’s *χ^2^* test for categorical data allowed for comparative evaluations. Comparisons between matched samples were carried out using the Wilcoxon signed-rank test or the McNemar test. We conducted a preliminary multivariate analysis of each lymphocyte subset count by fitting a linear generalized model on their logarithmic (log) values before booster dosing as a function of predefined covariates. A correlation between the log counts of lymphocyte subsets and either antibody titers or the length of V-TTF and OS was tested using the Spearman method. Based on a receiver operating characteristic (ROC) curve calculated at both time points, we evaluated the sensitivity and specificity of lymphocyte subpopulations in predicting the likelihood of DCB. The Youden index was applied to determine the optimal cut-point. For subsequent analyses, we deemed immune parameters relevant if they showed a statistically significant association with the intended outcome. A univariate analysis of the correlation between clinical variables and DCB was performed using Fisher’s exact test. A multivariate logistic regression model was implemented to estimate the odds ratio (OR) of DCB with a 95% CI in relation to the significant variables at univariate analysis. According to significant variables, a Mantel–Cox log-rank test allowed for comparison of survival outcomes between patient subgroups. The Kaplan–Meier method was used to visualize survival curves. To calculate the hazard ratio (HR) with a 95% CI of confirmed significant variables, a multivariate Cox regression model was applied. The tests were all two-sided, and a significant *p* value was defined as less than 0.05. All statistical evaluations and figure rendering were performed using SPSS (IBM SPSS Statistics for Windows, version 23.0, Armonk, NY, USA) and Prism (GraphPad, version 9), respectively.

## 3. Results

### 3.1. Patient Characteristics and General Outcomes

A total of 56 patients met inclusion criteria and were enrolled in the current subgroup analysis. All participants received a third dose of tozinameran between 23 September and 7 October 2021. At the time of the booster dosing, their median age was 66 years, and the majority of them (71%) were male. All recipients reported having a metastatic disease stage and an Eastern Cooperative Oncology Group Performance Status (ECOG PS) of 0 to 1. Non-small-cell or small-cell lung cancer was the most common diagnosis (66%). Pembrolizumab (45%) and nivolumab (37%) were the immune checkpoint inhibition treatments that were most frequently used. The median length of ICI treatment before and after the booster immunization was 8.4 and 4.6 months, respectively. At the time of the interim analysis, 11 (20%) patients were still being treated, 45 (80%) withdrew from treatment, and 19 (34%) were censored because no survival-relevant events occurred. At disease restaging performed within 6 months after the 3rd dose of tozinameran, we described 12 partial responses (21%), 23 disease stabilizations (41%), and 21 disease progressions (37%). Since all objective responses and 14 (25%) of disease stabilizations lasted longer than 6 months, we were able to report DCB and NCB in 26 (46%) and 30 (54%) patients, respectively. The general population had a median OS of 21.9 months (95% CI 14.8–26.2) after a median follow-up period of 32.3 months (95% CI 27.4–31.7). Table 1 depicts in detail the baseline characteristics of the enrolled patients.

### 3.2. Variations in Absolute Counts of Circulating Lymphocytes

**Figure 1:**
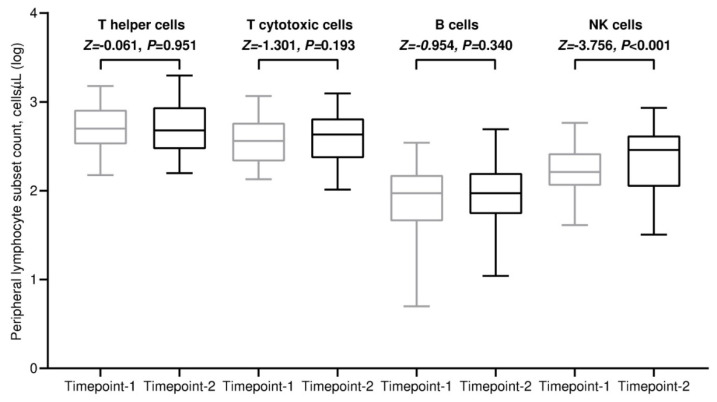
Dynamic changes in absolute counts of peripheral lymphocyte subpopulations. Bars denote median values with 95% confidence intervals. Differences between groups were assessed using the Wilcoxon signed-rank test. A two-sided *p* value < 0.05 was considered statistically significant. Log—logarithmic; T helper cells—CD3^+^CD4^+^ cells; T cytotoxic cell—CD3^+^CD8^+^; B cells—CD19^+^; NK—Natural killer, CD56^+^CD16^+^. Timepoint-1 indicates assessment before the third dose of tozinameran; timepoint-2 indicates assessment four weeks after the third dose of tozinameran.

All included patients completed a flow cytometry assessment of the peripheral blood at both time points. The absolute counts of each lymphocyte subpopulation showed notable variability among participants, which was even more pronounced after the third dose of tozinameran. The median values of all lymphocyte subpopulations increased after the third immunization in comparison with baseline. However, this incremental variation was statistically significant only for the NK cells (Figure 1 and Supplementary Table S1). The majority of patient subgroups experienced a significant increase in NK cell subpopulations after the third dose of tozinameran (Supplementary Table S2). It is worth noting that recipients with PD-L1-positive TPS reported the most prominent incremental change in NK cell counts (Supplementary Figure S2). We performed a multivariate analysis to verify whether predefined clinical variables affect lymphocyte subset counts across both time points. Among the most relevant findings, corticosteroid therapy at an immunosuppressive dosage was found to correlate with lower B cell counts both before and after the booster vaccination. In addition, the combination of ICIs and chemotherapy independently predicted decreased levels of T helper and T cytotoxic cells only at timepoint-2. No clinical variables had any significant impact on NK cell counts (Supplementary Tables S3 and S4). As a result of the third dose of tozinameran, anti-RBD-S1 IgG titers increased exponentially, with a median value of 714 BAU/mL (95% CI 179–1271) that was significantly higher than the same estimate obtained shortly before the booster vaccination [32 BAU/mL (95% CI 21–46), *p* < 0.001; Supplementary Figure S3]. Correlation analysis between anti-RBD-S1 IgG titers and lymphocyte counts did not detect any significant association at the assessment preceding the third dose of tozinameran (Supplementary Figure S4). The same testing performed after booster dosing showed a strong positive correlation for the NK cell subset, which was statistically significant [ρ = 0.47 (95% CI 0.22 to 0.66), *p* < 0.001; Supplementary Figure S5].

### 3.3. Clinical Benefit Outcome

**Figure 2:**
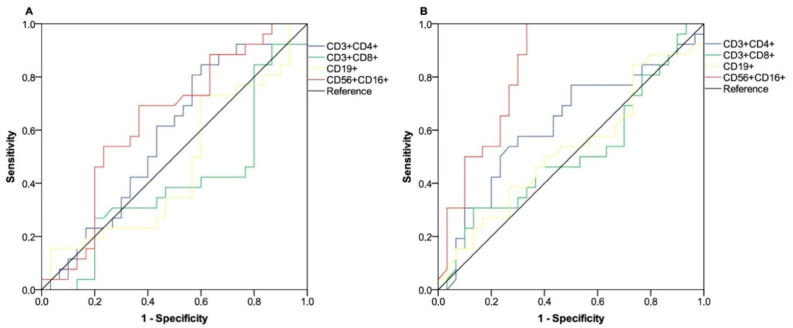
ROC curve analysis of peripheral lymphocyte counts on clinical benefit outcome.

To determine the relationship between absolute counts of peripheral lymphocyte subpopulations and DCB, a receiver operating characteristic (ROC) curve was computed at both time points. Relative values of the area under the curve (AUC) pertaining to lymphocyte distributions were not considered viable in predicting the probability of a positive outcome at the first time point (Figure 2A). Conversely, the subset of NK cells tested after the third dose of tozinameran revealed a significant association with DCB and was therefore considered relevant for subsequent evaluations [AUC 0.83 (95% CI 0.72–0.94), *p* < 0.001; Figure 2B]. The Youden index identified a count of 222/µL as the optimal cut-point for NK cell distribution. This threshold value yielded a sensitivity of 0.88 and a specificity of 0.70, allowing recipients to be divided into distinct subgroups of low-responders (low-R, <222 BAU/µL) and high-responders (high-R, ≥222/µL). As expected, univariate analysis showed a statistically significant association between DCB and several clinical or pathological variables, including a diagnosis differing from lung cancer, a low disease burden, lack of bone or liver involvement, a weight loss ≤ 10%, and positive PD-L1 expression. The high-responder subgroup also had a better outcome in the same comparison (*p* < 0.001). As a result of a multivariate analysis, the number of metastatic sites, PD-L1 expression, and NK cell level after the booster dose remained significant predictors (Table 2). Of note, after a median follow-up of 226 days (95% CI 125–525), 13 patients in the low-responder subgroup (50%) and 7 in the high-responder subgroup (23.3%, *p* = 0.037) reported contracting SARS-CoV-2 infection, none of which was clinically severe.

Receiver operating characteristic (ROC) curves are represented for subsets of peripheral lymphocytes. (A) ROC analysis showing the performance of absolute counts of peripheral lymphocyte subsets in predicting durable clinical benefit at timepoint-1. AUC for the subpopulation relative values: T helper cells (CD3^+^CD4^+^): 0.58 (95% CI 0.43–0.73; *p* = 0.301); T cytotoxic cells (CD3^+^CD8^+^): 0.40 (95% CI 0.24–0.55; *p* = 0.203); B cells (CD19^+^): 0.64 (95% CI 0.30–0.62; *p* = 0.646); NK cells (CD56^+^CD16^+^): 0.63 (95% CI 0.49–0.78; *p* = 0.077). (B) ROC analysis showing the performance of absolute counts of peripheral lymphocyte subsets in predicting durable clinical benefit at timepoint-2; AUC for the subpopulation relative values: T helper cells (CD3^+^CD4^+^): 0.62 (95% CI 0.47–0.77; *p* = 0.115); T cytotoxic cells (CD3^+^CD8^+^): 0.51 (95% CI 0.35–0.66; *p* = 0.889); B cells (CD19^+^): 0.52 (95% CI 0.37–0.68; *p* = 0.718); NK cells (CD56^+^CD16^+^): 0.83 (95% CI 0.72–0.94; *p* < 0.001). AUC—area under the curve; NK—Natural Killer; CI—confidence interval. Timepoint-1 indicates assessment before the third dose of tozinameran; timepoint-2 indicates assessment four weeks after the third dose of tozinameran.

### 3.4. Survival Outcome

**Figure 3:**
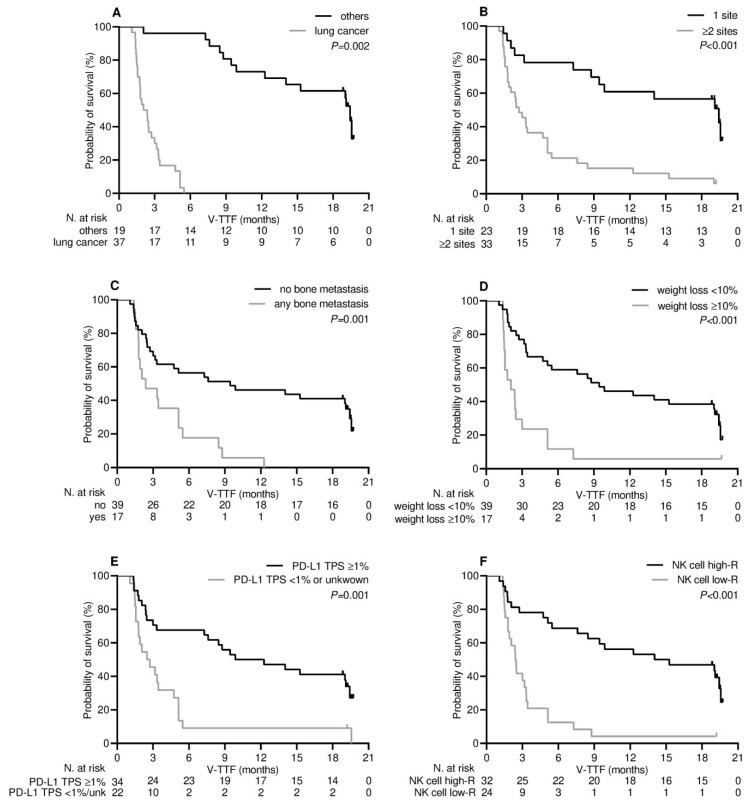
Vaccine-related time-to-treatment failure depending on significant clinical variables. (**A**) Type of cancer diagnosis: others vs. lung cancer; (**B**) metastatic extent of disease: 1 site vs. ≥2 sites; (**C**) bone metastatic involvement: not present vs. any; (**D**) weight loss from baseline: <10% vs. ≥10%; (**E**) programmed cell death-ligand 1 tumor proportion score (PD-L1 TPS): >1% vs. <1% or unknown; (**F**) level of NK cell response: high-R (subgroup of patients with NK cell count ≥ 222/µL after the third dose of tozinameran) vs. low-R (subgroup of patients with NK cell count < 222/µL after the third dose of tozinameran).

**Figure 4:**
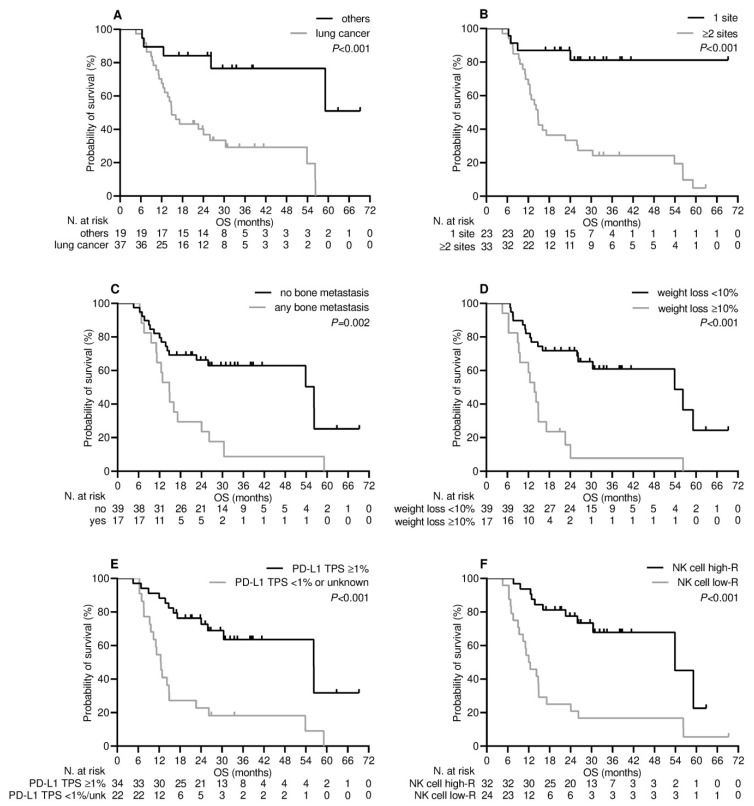
Overall survival depending on significant clinical variables. (**A**) Type of cancer diagnosis: others vs. lung cancer; (**B**) metastatic extent of disease: 1 site vs. ≥2 sites; (**C**) bone metastatic involvement: not present vs. any; (**D**) weight loss from baseline: <10% vs. ≥10%; (**E**) programmed cell death-ligand 1 tumor proportion score (PD-L1 TPS): >1% vs. <1% or unknown; (**F**) level of NK cell response: high-R (subgroup of patients with NK cell count ≥ 222/µL after the third dose of tozinameran) vs. low-R (subgroup of patients with NK cell count < 222/µL after the third dose of tozinameran).

**Figure 5:**
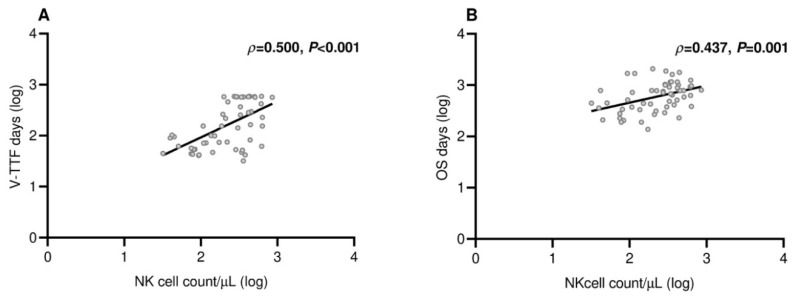
Scatter plot of survival by NK cell counts. (**A**) Vaccine-related time-to-treatment failure: ρ = 0.500 (95% CI 0.279–0.667), *p* < 0.001; (**B**) overall survival: ρ = 0.437 (95% CI 0.219–0.619), *p* = 0.001. Log—logarithmic values; CI—confidence interval.

Patients who experienced clinical benefit six months after receiving the third dose of tozinameran experienced a significant improvement in their median V-TTF and OS. Supplementary Figure S6A shows that the median V-TTF in the DCB group was 19.4 months (95% CI 18.7–20.1) as opposed to 2.0 months for patients in the NCB subgroup (95% CI 1.2–2.8, *p* < 0.001). Likewise, as shown in Supplementary Figure S6B, the median OS for the DCB subgroup was 38.0 months (95% CI 31.5–44.5) as opposed to 12.5 months (95% CI 10.1–14.9) for patients in the NCB subgroup (*p* < 0.001). Based on this finding, variables related to DCB are likely to affect V-TTF and OS [19]. The results of univariate survival testing consistently confirmed that the variables identified as significantly associated with DCB in the multivariate analysis were reliable survival predictors. All of these covariates were associated with a decreased risk of loss of both clinical benefit (Table 3 and Figure 3) and mortality (Table 3 and Figure 4). However, in the multivariate analysis, only NK cell count levels after booster dosing retained an independent impact on both V-TTF and OS (Table 3). In addition, there was a positive linear correlation for either V-TFF [ρ = 0.50 (95% CI 0.29–0.67), *p* < 0.001; Figure 5A] or OS [ρ = 0.43 (95% CI 0.17–0.63), *p* = 0.001; Figure 5B] when evaluating survival outcomes in relation to log values of NK cell counts.

## 4. Discussion

In both healthy and immunocompromised recipients, the relationship between anti-SARS-CoV-2 mRNA vaccination and systemic immunity shows a complex interplay that results in clinically relevant effects [20]. In cancer patients receiving immune checkpoint blockade, the implications for innate and adaptive cell-mediated responses may be even more pronounced [21]. A SARS-CoV-2 transmembrane spike protein that is specifically encoded by COVID-19 mRNA-based vaccines may have pro-inflammatory effects in human tissues due to its shedding and binding to angiotensin-converting enzyme 2 [22]. Conversely, blocking the PD-1/PD-L1 signaling pathway may cause the reactivation of T cells that are specific for the SARS-CoV-2 spike protein, increasing cytokine release and resulting in clinical events [23].Concurrent treatment with ICIs has not been shown to increase the risk of developing severe immune-related adverse events after booster doses of the COVID-19 vaccine in a series of retrospective studies [24,25,26,27]. However, it is still unclear how post-mRNA vaccination changes in cellular immunity affect the clinical activity of ICIs. There have been concerns about a rise in tumor hyper-progression brought on by increased immunogenicity and T cell response stimulation [28]. Additional evidence points to mRNA immunization itself as a trigger for biological processes that, in various ways, are detrimental to the maintenance of immune competence and cellular homeostasis [29]. The suppression of IFNα and its downstream signaling cascade impairs cancer immunoediting, which could potentially interfere with immune checkpoint blockade [30]. However, the question whether enhanced effects of the booster vaccination have a functional impact on the efficacy of ICIs still remains unaddressed [31].

In this research, we investigated dynamic changes in absolute counts of circulating lymphocytes and disease outcomes of patients treated with anti-PD-1/PD-L1 agents for a broad spectrum of advanced solid tumors over an 18-month time frame. Although lengthwise assessment did not reveal associations with lymphocyte subpopulations before the third dose, we observed a significant positive correlation with NK cell count after the booster immunization. It is worth noting that recipients who had a more sustained NK cell response also appeared to derive better survival outcomes due to a significantly reduced likelihood of treatment failure and death. To the best of our knowledge, similar results have not been previously reported and warrant a critical appraisal of their relevance from both methodological and clinical perspectives. The reliability of using absolute counts of circulating lymphocyte subsets as a correlate of adaptive immunity induced by mRNA-based vaccination is still a matter of debate. This approach has inherent strengths and weaknesses. In cancer patients vaccinated against SARS-CoV-2, the cell-mediated immune response has been assessed through enzyme-linked immunosorbent spot (ELISpot) tests to identify IFNγ-producing specific T cells [32,33]. Despite their high accuracy, the lack of standardization and methodological challenges still hinder the broad implementation of these assays. Immunophenotypic characterization of peripheral blood provides a generic depiction of lymphocyte dynamics following the third dose of tozinameran. Nonetheless, several studies have observed that the results of SARS-CoV-2-specific T and B cell assays and their absolute counts are in close agreement, which supports the viability of this methodology in tracking adaptive immunity in the context of COVID-19 vaccination [34,35]. Furthermore, changes in peripheral lymphocyte subpopulations may also reflect the effects of vaccine-independent variables, including dynamic interactions between tumor microenvironment and immune checkpoint blockade [36]. Although there was a significant impact of cytotoxic chemotherapy on T cell counts after the booster dose, a multivariate analysis ruled out biased patient selection as a result of confounding effects of the remaining clinical and pathological variables.

We observed a significant incremental change in absolute NK cell counts 28 days after the third dose of tozinameran. This dynamic variation had a positive correlation with an enhanced humoral response. Of note, the rate of SARS-CoV-2 breakthrough infections was significantly lower in recipients with an increased NK cell response. NK cells are the most important components of innate immunity and can be primarily classified into two subgroups based on their surface densities of CD56 and CD16 [37]. The CD56^dim^CD16^bright^ subset exerts a more pronounced natural cytotoxicity with prevalent tissue localization. Conversely, the CD56^bright^CD16^dim^ subset exhibits lower cytotoxic properties but retains the ability to produce cytokines and chemokines upon monocyte activation. Since the latter subpopulation is predominant in peripheral blood, it largely matches the NK cell subset captured by our gating strategy [38]. Accordingly, our results are consistent with current evidence suggesting a contribution of early NK cell activation to the emergence of immunity against SARS-CoV-2 after immunization with an mRNA-based vaccine [9,39,40]. We next investigated the impact of changes in absolute NK cell counts following the booster dose of tozinameran on disease outcomes. Patients who exhibited an incremental variation in this subpopulation of lymphocytes had a significantly reduced likelihood of disease progression within six months of vaccination and longer overall survival. NK cells play an essential role in the development of anti-cancer immunity [41]. The tumor microenvironment induces NK cell dysfunction through enhanced expression of inhibitory immune checkpoints and downregulation of activating receptors [42]. Several solid malignancies promote PD-1 expression on circulating and tumor-infiltrating NK cells with adverse prognostic implications [43]. In addition to its central role in triggering T-cell responses, modulation of co-inhibitory PD-1/PD-L1 signaling pathway on NK cells can provide a viable contribution to the success of immunotherapies based on immune checkpoint blockade. Restoration of NK cell functions has been suggested to improve the therapeutic index of cancer immunotherapy [44]. In this regard, a series of pivotal investigations support the efficacy of novel agents targeting NK cell-specific immune checkpoint molecules at preclinical and early clinical levels [45]. At the same time, several studies have already shown that increases in peripheral frequencies of these immune cell fractions correlate with improved survival during treatment with currently available ICIs [46,47,48]. Disease outcomes observed in the current research appear to be roughly consistent with the findings of these studies. Furthermore, multivariable and correlation analyses with survival length indicate a favorable interaction between the effects of an additional immunization on NK cell frequencies and the efficacy of ICIs. Activation of circulating NK cells, as occurs after a booster dose of mRNA-based vaccines, leads to increased IFNγ production [49]. This humoral event is widely known for its critical role in modulating innate and adaptive immune responses mediated by T helper and T cytotoxic lymphocytes against several conditions, including the development of cancer cells [50]. Since changes in absolute counts of peripheral NK cells have been considered viable correlates of IFNγ-specific responses, this evidence could underlie the clinical relevance of our findings [51]. According to two retrospective studies, influenza vaccination even resulted in a survival benefit during ICI exposure, excluding the putative risk of hyper-progression commonly associated with antiviral immunization [52,53]. There is also evidence that camrelizumab, an alternative monoclonal antibody targeting the PD-1 checkpoint, may improve clinical outcomes in patients receiving the SARS-CoV-2 BBIPB-CorV vaccine compared with their unvaccinated counterparts [54]. Regardless of the differences in research methodologies, our results seem to support the evidence that immunization against SARS-CoV-2 infection does not blunt the effectiveness of immune checkpoint blockade.

This study recognizes additional limitations beyond the previous methodological issues. We recruited a large sample of patients over a short time frame in response to the need for managing the emergency associated with the COVID-19 outbreak. This approach ruled out the possibility of proper stratification of participants, making the study susceptible to selection imbalances. Our research covered a broad spectrum of solid tumors, indicating that the effects of vaccination and immunotherapies may differ among people with diverse types of cancer diagnoses. The design of this study has an implicit immortal-temporal bias, which results from the gap in time between starting immunotherapy and vaccination, potentially causing the survival benefit to be overstated [55]. The choice of V-TTF as a surrogate survival endpoint, which refers to a homogeneous event over time for all patients, may mitigate but not eliminate the effects of this potential unbalance. In addition, we did not provide an independent review of imaging or its reassessment according to immune-related criteria for treatment response [56]. These drawbacks may have led to an inaccurate appraisal of anti-PD-1/PD-L1 blockade activity and V-TTF length. Lastly, the size of our study population is small, as is the duration of follow-up after the third dose of the vaccine. This further suggests that multivariate statistical analyses might generate false-positive results, and their significance should therefore be regarded as exploratory.

## 5. Conclusions

In advanced cancer patients receiving ICIs, many areas of uncertainty remain concerning the clinical consequences of booster doses of mRNA-based immunization. Because COVID-19 vaccination may elicit highly unpredictable immune responses, the potential for an adverse impact on disease outcomes cannot be neglected. We prospectively investigated the effects of peripheral immune cell dynamics in this condition for the first time. As in previous studies, no cases of hyper-progression were observed, nor did the loss of clinical rate benefit vary from what was expected before the third dose of tozinameran. We also described a significant survival gain in favor of recipients with an incremental change in circulating NK cell counts, which could strengthen previous suggestions. Mechanistically, this finding could also indicate a favorable interaction at the immunological level. However, the lack of comparable studies, as well as the impossibility of comparison with a control group of unvaccinated patients, does not allow further insights to be drawn. This study has inherent limitations, suggesting that its results may only generate hypotheses that warrant further investigation in larger independent series.

## Figures and Tables

**Table 1:** Patient characteristics.

Characteristic	General Population, N = 56 (100%)
Mean age, years (SD)	65.9 (10.0)
Sex	
- female	16 (28.6%)
- male	40 (71.4%)
ECOG PS	
- 0	17 (30.4%)
- 1	39 (69.6%)
Cancer type	
- non-small-cell lung cancer	31 (55.3%)
- small-cell lung cancer	6 (10.7%)
- kidney	7 (12.5%)
- dermal (melanoma, Merkel-cell, squamous cell carcinoma)	7 (12.5%)
- bladder	4 (7.1%)
- esophageal	1 (1.8%)
Disease extent	
- metastatic	56 (100%)
Treatment setting	
- metastatic, first line	36 (64.3%)
- metastatic, second or later line	20 (35.7%)
Number of metastatic sites	
- 1	23 (41.1%)
- ≥2	33 (58.9%)
Brain metastases	
- not present	44 (78.6%)
- any	12 (21.4%)
Liver metastases	
- not present	50 (89.3%)
- any	6 (10.7%)
Bone metastases	
- not present	39 (69.6%)
- any	17 (30.4%)
Weight loss ^a^	
- <10%	39 (69.6%)
- ≥10%	17 (30.4%)
PD-L1 TPS	
- ≥1%	35 (62.5%)
- <1%	16 (28.5%)
- unknown	5 (8.9%)
Corticosteroid therapy ^b^	8 (14.3%)
ICI-based treatment	
- pembrolizumab	25 (44.6%)
- nivolumab	21 (37.5%)
- atezolizumab	6 (10.7%)
- durvalumab	2 (3.6%)
- avelumab	1 (1.8%)
- cemiplimab	1 (1.8%)
Length (months) of ICI treatment, median (IQR)	19.8 (10.5–26.9)
Length (months) of ICI treatment before third vaccine dose, median (IQR)	8.4 (5.1–16.1)
Length (months) of ICI treatment after third vaccine dose, median (IQR)	4.6 (1.5–18.8)

SD—standard deviation; ECOG PS—Eastern Cooperative Oncology Group Performance Status; PD-L1 TPS—programmed cell death-ligand 1 tumor proportion score; ICI—immune checkpoint inhibitor; IQR—interquartile range. ^a^ weight loss indicates body weight variation in the 90 days preceding the third dose of tozinameran; ^b^ corticosteroid therapy indicates ≥10 mg prednisone equivalent daily for at least 7 days in the 28 days preceding the third dose of tozinameran.

**Table 2:** Analysis of clinical benefit outcome.

	Univariate Analysis			Multivariate Analysis	
	NCB N = 30 (100%)	DCB N = 26 (100%)	*p* Value	OR (95% CI)	*p* Value
Age			0.99	-	-
- ≤70 years (N = 32)	17 (56.7%)	15 (57.7%)			
- >70 years (N = 24)	13 (43.3%)	11 (42.3%)			
Sex			0.55	-	-
- female (N = 16)	10 (33.3%)	6 (23.1%)			
- male (N = 40)	20 (66.7%)	20 (76.9%)			
ECOG PS			0.25	-	-
- 0 (N = 17)	7 (23.3%)	10 (38.5%)			
- 1 (N = 39)	23 (76.7%)	16 (61.5%)			
Cancer type			0.005^ †^		0.16
- any other (N = 19)	5 (16.7%)	14 (53.8%)		1.00	
- lung (N = 37)	25 (83.3%)	12 (46.2%)		0.25 (0.03–1.71)	
Number of metastatic sites			<0.001^ †^		0.026
- 1 (N = 23)	4 (13.3%)	19 (73.1%)		1.00	
- ≥2 (N = 33)	26 (86.7%)	7 (26.9%)		0.10 (0.01–0.76)	
Brain metastases			0.34	-	-
- not present (N = 44)	22 (73.3%)	22 (84.6%)			
- any (N = 12)	8 (26.7%)	4 (15.4%)			
Bone metastases			0.008^ †^		0.66
- not present (N = 39)	16 (53.3%)	23 (88.5%)		1.00	
- any (N = 17)	14 (46.7%)	3 (11.5%)		0.68 (0.12–3.85)	
Liver metastases			0.025	-	-
- not present (N = 50)	24 (80.0%)	26 (100%)			
- any (N = 6)	6 (20.0%)	-			
Weight loss ^a^			0.001^ †^		0.28
- <10% (N = 39)	15 (50.0%)	24 (92.3%)		1.00	
- ≥10% (N = 17)	15 (50.0%)	2 (7.7%)		0.31 (0.03–2.65)	
PD-L1 TPS			<0.001^ †^		0.010
- <1% or unknown (N = 22)	20 (66.7%)	2 (7.7%)		1.00	
- >1% (N = 34)	10 (33.3%)	24 (92.3%)		13.29 (1.86–94)	
Treatment setting			0.26	-	-
- first line (N = 36)	17 (56.7%)	19 (73.1%)			
- second or later line (N = 20)	13 (43.3%)	7 (26.9%)			
Corticosteroid therapy ^b^			0.34	-	-
- no (N = 44)	22 (73.3%)	22 (84.6%)			
- yes (N = 12)	8 (26.7%)	4 (15.4%)			
ICI therapy			0.48	-	-
- anti-PD-1 (N = 47)	24 (80.0%)	23 (88.5%)			
- anti-PD-L1 (N = 9)	6 (20.0%)	3 (11.5%)			
Treatment type			0.14	-	-
- ICI monotherapy	18 (60.0%)	21 (80.8%)			
- ICI and chemotherapy	12 (40.0%)	5 (19.2%)			
NK cell level ^c^			<0.001^ †^		0.020
- low-responders (N = 24)	21 (70.0%)	3 (11.5%)		1.00	
- high-responders (N = 32)	9 (30.0%)	23 (88.5%)		12.31 (1.48–102)	

NCB—no clinical benefit; DCB—durable clinical benefit; OR—odds ratio; CI—confidence interval; ECOG PS—Eastern Cooperative Oncology Group Performance Status; PD-L1 TPS—programmed cell death-ligand 1 tumor proportion score; ICI—immune checkpoint inhibitor. ^a^ weight loss indicates body weight change during 90 days before the third dose of tozinameran; ^b^ corticosteroid therapy indicates ≥ 10 mg prednisone equivalent daily for at least 7 days during 28 days before the third dose of tozinameran; ^c^ low-responders indicate the subgroup of patients with NK cell count < 222/µL after the third dose of tozinameran, high-responders indicate the subgroup of patients with NK cell count ≥ 222/µL after the third dose of tozinameran; ^†^ statistical significance maintained after Holm–Bonferroni *p* value correction for multiple comparisons.

**Table 3:** Analysis of survival outcomes.

Variable	Vaccine-Related Time-to-Treatment Failure				Overall Survival			
	Univariate Analysis		Multivariate Analysis		Univariate Analysis		Multivariate Analysis	
	Median V-TTF (95% CI), Months	*p* Value	HR (95% CI)	*p* Value	Median OS (95% CI), Months	*p* Value	HR (95% CI)	*p* Value
Cancer type		0.002		0.091		<0.001		0.019
- others	19.0 (10.5–27.5)		1.00		38.0 (27.5-NR)		1.00	
- lung	2.7 (1.8–3.6)		1.96 (0.89–4.29)		14.9 (11.4–18.3)		4.18 (1.26–13.86)	
Number of metastatic sites		<0.001		0.007		<0.001		0.14
- 1	19.4 (10.5–28.2)		1.00		26.2 (23.8-NR)		1.00	
- ≥2	2.7 (1.7–3.7)		3.08 (1.36–6.99)		14.7 (12.4–17.0)		2.37 (0.73–7.63)	
Bone metastases		0.001		0.47		0.002		0.50
- not present	9.4 (0.1–9.3)		1.00		56.2 (21.8–90.6)		1.00	
- any	2.3 (0.4–4.2)		1.30 (0.63–2.68)		14.8 (11.5–18.1)		1.31 (0.58–2.93)	
Weight loss ^a^		<0.001		0.47		<0.001		0.83
- <10%	9.4 (3.7–15.1)		1.00		53.8 (27.2–80.4)		1.00	
- ≥10%	2.0 (1.0–3.1)		1.34 (0.60–2.98)		13.7 (10.7–16.7)		1.09 (0.46–2.58)	
PD-L1 TPS		0.001		0.47		<0.001		0.015
- <1% or unknown	2.5 (1.0–3.9)		1.00		12.4 (10.3–14.5)		1.00	
- ≥1%	9.8 (2.3–17.3)		0.76 (0.37–1.59)		56.2 (31.7–80.7)		0.34 (0.14–0.81)	
NK cell level ^b^		<0.001		0.014		<0.001		0.027
- low-responders	2.4 (1.8–2.9)		1.00		12.0 (8.1–15.8)		1.00	
- high-responders	14.0 (1.8–26.2)		0.34 (0.14–0.80)		56.8 (29.6–78.0)		0.36 (0.15–0.89)	

HR—hazard ratio; CI—confidence interval; PD-L1 TPS—programmed cell death-ligand 1 tumor proportion score; NR—not reached; ^a^ weight loss indicates body weight variation in the 90 days preceding the third dose of tozinameran; ^b^ low-responders indicate the subgroup of patients with NK cell count <222/µL after the third dose of tozinameran, high-responders indicate the subgroup of patients with NK cell count ≥222/µL after the third dose of tozinameran.

## Data Availability

The datasets generated and analyzed during the current study are available from the corresponding author on reasonable request.

## Supplementary Materials

The following supporting information can be downloaded at: https://www.mdpi.com/article/10.3390/cancers15143625/s1, Supplementary Table S1: Univariate analysis of dynamic changes in peripheral lymphocyte counts; Supplementary Table S2: Univariate analysis of dynamic changes in peripheral lymphocyte counts by predefined clinical variables; Supplementary Table S3: Multivariate analysis of peripheral lymphocyte counts by predefined clinical variables at timepoint-1; Supplementary Table S4: Multivariate analysis of peripheral lymphocyte counts by predefined clinical variables at timepoint-2; Supplementary Figure S1: Flow cytometry analysis; Supplementary Figure S2: Dynamic changes in absolute counts of peripheral lymphocyte subpopulations by PD-L1 TPS; Supplementary Figure S3: Longitudinal comparison of scatter plot distributions and medians of antibody titers; Supplementary Figure S4: Correlation between antibody titers and lymphocyte subpopulation counts before the third dose of tozinameran; Supplementary Figure S5: Correlation between antibody titers and lymphocyte subpopulation counts after the third dose of tozinameran; Supplementary Figure S6: Time-to-event depending on clinical benefit outcome.
