# Supplementary material for: Changes in Peripheral Immune Cells after the Third Dose of SARS-CoV-2 mRNA-BNT162b2 Vaccine and Disease Outcomes in Cancer Patients Receiving Immune Checkpoint Inhibitors: A Prospective Analysis of the Vax-on-Third-Profile Study
Source: Cancers (Basel). 2023 Jul 14;15(14):3625. doi: 10.3390/cancers15143625 (PMC10377319; doi:10.3390/cancers15143625)
Supplement: Supplementary file 1 [file cancers-15-03625-s001.zip › cancers-2472975-supplementary.pdf]

SUPPLEMENTARY MATERIAL

Supplementary Table S1. Univariate analysis of dynamic changes in peripheral lymphocyte counts

Lymphocyte subpopulation (absolute count/μL), median with 95% CI	Timepoint-1	Timepoint-2	P value
T helper cells	500 (397-664)	508 (406-694)	0.95
T cytotoxic cells	364 (297-509)	429 (339-520)	0.19
B cells	94 (62-116)	96 (67-130)	0.34
NK cells	162 (137-215)	287 (190-343)	**<0.001**

Statistically significant *P* values are highlighted in bold. *P* values derived from Wilcoxon signed-rank test for pairwise comparisons between timepoint-1 and timepoint-2. A two-sided P value of <0.05 was considered statistically significant. CI, confidence intervals; T helper cells, $CD3^{+}CD4^{+}$ cells; T cytotoxic cell, $CD3^{+}CD8^{+}$; B cells, $CD19^{+}$; NK, Natural killer, $CD56^{+}CD16^{+}$; Timepoint-1 denotes assessment before the third dose of tozinameran.

Supplementary Table S2. Univariate analysis of dynamic changes in peripheral lymphocyte counts by predefined clinical variables

Covariate	T helper cells (absolute count/μL), median with 95% CI		P value	T cytotoxic cells (absolute count/μL), median with 95% CI		P value	B cells (absolute count/μL), median with 95% CI		P value	NK cells (absolute count/μL), median with 95% CI		P value
	Timepoint-1	Timepoint-2		Timepoint-1	Timepoint-2		Timepoint-1	Timepoint-2		Timepoint-1	Timepoint-2	
Sex												
- female	610 (459-789)	597 (401-818)	0.60	391 (300-490)	464 (327-615)	0.32	120 (82-1654)	136 (92-180)	0.79	171 (123-223)	258 (170-363)	0.05
- male	619 (499-747)	653 (526-794)	0.70	435 (362-508)	482 (388-570)	0.34	103 (79-129)	115 (87-143)	0.31	215 (179-252)	302 (243-360)	**0.001**
Age (years)												
- ≤70	560 (451-682)	580 (440-745)	0.88	403 (331-479)	454 (354-561)	0.43	116 (87-146)	130 (96-169)	0.27	189 (157-226)	275 (217-336)	**0.002**
- >70	692 (546-846)	714 (544-887)	0.72	449 (351-556)	508 (387-647)	0.24	97 (70-125)	109 (80-140)	0.79	220 (169-280)	307 (219-386)	**0.022**
Cancer type												
- others	650 (507-799)	723 (528-931)	0.26	384 (293-480)	490 (371-624)	0.05	109 (78-142)	139 (96-189)	0.20	199 (152-241)	333 (262-399)	**0.002**
- lung	599 (478-722)	593 (463-738)	0.43	443 (161-516)	470 (372-581)	0.89	107 (81-132	111 (86-138)	0.88	204 (167-247)	267 (208-339)	**0.035**
ECOG PS												
- 0	800 (619-998)	803 (555-1068)	0.83	452 (328-601)	484 (356-621)	0.52	140 (99-188)	160 (107-226)	0.40	236 (181-295)	326 (229-415)	0.05
- 1	536 (446-637)	565 (466-685)	0.83	410 (351-475)	474 (376-581)	0.21	94 (75-115)	104 (81-128)	0.57	188 (156-225)	273 (215-339)	**0.001**
Number of metastatic sites												
- 1	707 (551-869)	724 (543-903)	0.82	425 (327-535)	489 (363-613)	0.16	120 (82-162)	123 (81-173)	0.87	240 (191-294)	377 (302-462)	**0.001**
- ≥2	553 (455-674)	577 (426-733)	0.85	421 (349-496)	469 (367-577)	0.84	99 (79-120)	119 (91-150)	0.28	176 (141-214)	228 (172-288)	**0.030**
Brain metastases												
- absent	639 (525-753)	659 (524-800)	0.98	417 (353-490)	472 (381-573)	0.17	107 (86-132)	117 (89-149)	0.58	217 (181-256)	306 (249-363)	**0.001**
- any	536 (410-676)	559 (383-752)	0.75	444 (349-538)	497 (540-678)	0.87	112 (68-155)	135 (87-183)	0.27	150 (111-190)	226 (131-333)	**0.023**
Bone metastases												
- absent	631 (521-738)	669 (543-789)	0.65	424 (356-500)	509 (412-605)	0.09	106 (83-131)	121 (92-155)	0.39	229 (191-268)	334 (277-391)	**<0.001**

- any	582 (411-783)	563 (345-847)	0.43	420 (330-516)	405 (289-536)	0.70	113 (75-155)	120 (82-166)	0.65	142 (107-183)	186 (116-270)	0.36
Liver metastases												
- absent	638 (538-735)	658 (538-790)	0.96	425 (360-495)	470 (390-560)	0.32	104 (84-128)	118 (92-146)	0.39	212 (180-247)	306 (251-361)	**<0.001**
- any	441 (303-655)	461 (301-658)	0.75	406 (329-494)	535 (371-821)	0.14	134 (79-187)	141 (101-174)	0.60	124 (96-161)	144 (86-233)	0.60
PD-L1 TPS												
- >1%	652 (539-777)	689 (561-828)	0.70	407 (338-491)	473 (383-576)	0.14	112 (84-142)	128 (95-164)	0.42	214 (173-261)	335 (277-395)	**<0.001**
- <1% or unknown	562 (426-725)	558 (385-759)	0.63	447 (348-543)	483 (357-616)	0.83	101 (73-130)	109 (78-141)	0.53	184 (142-225)	218 (149-288)	0.46
Treatment setting												
- 1st line	670 (552-816)	737 (580-897)	0.28	448 (366-530)	529 (440-623)	0.07	119 (92-145)	142 (107-178)	0.15	217 (177-256)	323 (255-391)	**0.001**
- 2nd or later line	520 (405-644)	458 (349-562)	0.09	377 (307-447)	383 (277-509)	0.57	87 (60-118)	83 (62-106)	0.62	176 (130-223)	227 (164-294)	0.07
Corticosteroid therapy												
- no	640 (530-747)	690 (557-829)	0.57	419 (352-490)	484 (395-576)	0.05	117 (95-142)	138 (111-168)	0.22	211 (176-252)	301 (247-361)	**0.001**
- yes	532 (349-724)	445 (307-599)	0.27	438 (323-563)	451 (306-628)	0.43	72 (33-121)	58 (37-81)	0.69	171 (128-214)	246 (144-358)	0.13
Time from last active treatment												
- ≤28 days	607 (478-737)	616 (466-781)	0.98	434 (341-543)	453 (354-562)	0.43	109 (79-141)	120 (85-158)	0.83	185 (143-229)	266 (197-337)	0.06
- >28 days	622 (495-756)	650 (499-813)	0.95	416 (345-491)	491 (385-605)	0.32	107 (82-137)	121 (91-158)	0.29	212 (172-257)	303 (238-373)	**0.001**
Treatment type												
- ICI	646 (540-754)	685 (564-820)	0.91	424 (355-499)	507 (411-607)	0.10	108 (83-134)	128 (100-161)	0.24	223 (183-266)	318 (256-379)	**0.001**
- Chemotherapy & ICI	548 (396-756)	527 (326-785)	0.98	420 (309-532)	409 (285-540)	0.67	108 (74-141)	103 (67-142)	1.00	155 (118-192)	222 (144-308)	0.05

Statistically significant *P* values are highlighted in bold. *P* values derived from Wilcoxon signed-rank test for pairwise comparisons between timepoint-1 and timepoint-2. A two-sided *P* value of <0.05 was considered statistically significant. Log, logarithmic values; CI, confidence intervals; ECOG PS, Eastern Cooperative Oncology Group Performance Status; PD-L1 TPS, programmed cell death-ligand 1 tumor proportion score; ICI, immune checkpoint inhibitor. Corticosteroid therapy indicates ≥10 mg prednisone equivalent daily for at least 7 days in the 28 days preceding the third dose of vaccine; T helper cells, $CD3^{+}CD4^{+}$ cells; T cytotoxic cell, $CD3^{+}CD8^{+}$; B cells, $CD19^{+}$; NK, Natural killer, $CD56^{+}CD16^{+}$; Timepoint-1 denotes assessment before the third dose of tozinameran.

Supplementary Table S3. Multivariate analysis of peripheral lymphocyte counts by predefined clinical variables at timepoint-1

Covariate	T helper cell count (log)		T cytotoxic cell count (log)		B cell count (log)		NK cell count (log)	
	Beta (95% CI)	P value	Beta (95% CI)	P value	Beta (95% CI)	P value	Beta (95% CI)	P value
Sex<br>- male vs female	-0.05 (-0.22 to 0.11)	0.502	0.04 (-0.12 to 0.20)	0.626	0.01 (-0.22 to 0.24)	0.956	0.02 (-0.12 to 0.18)	0.729
Age (years)<br>- >70 vs ≤70	0.12 (-0.02 to 0.28)	0.108	0.02 (-0.13 to 0.17)	0.796	-0.13 (-0.34 to 0.08)	0.229	0.01 (-0.13 to 0.15)	0.885
Cancer type<br>- lung vs others	0.02 (-0.13 to 0.18)	0.755	0.10 (-0.05 to 0.26)	0.197	0.02 (-0.20 to 0.24)	0.839	0.11 (-0.03 to 0.26)	0.125
ECOG PS<br>- 1 vs 0	-0.13 (-0.30 to 0.03)	0.117	-0.01 (-0.17 to 0.15)	0.922	-0.15 (-0.38 to 0.08)	0.202	-0.02 (-0.17 to 0.12)	0.733
Number of metastatic sites<br>- ≥2 vs 1	0.01 (-0.16 to 0.17)	0.949	-0.01 (-.17 to 0.16)	0.938	-0.13 (-0.37 to 0.10)	0.262	-0.05 (-0.20 to 0.10)	0.521
Brain metastases<br>- yes vs no	0.01 (-0.16 to 0.16)	0.996	0.04 (-0.11 to 0.21)	0.551	0.06 (-0.16 to 0.28)	0.600	-0.09 (-0.24 to 0.05)	0.216
Bone metastases<br>- yes vs no	-0.01 (-0.17 to 0.16)	0.949	0.02 (-0.14 to 0.18)	0.781	0.01 (-0.22 to 0.24)	0.930	-0.09 (-0.24 to 0.09)	0.210
Liver metastases<br>- yes vs no	-0.09 (-0.33 to 0.13)	0.407	0.03 (-0.19 to 0.26)	0.771	0.30 (-0.01 to 0.62)	0.065	-0.12 (-0.33 to 0.09)	0.270
PD-L1 TPS<br>- >1% vs <1% or unknown	0.03 (-0.12 to 0.19)	0.679	-0.02 (-0.18 to 0.13)	0.750	-0.17 (-0.40 to 0.04)	0.121	-0.07 (-0.21 to 0.11)	0.350
Metastatic treatment setting								

- first line vs second or later line	-0.08 (-0.27 to 0.09)	0.353	-0.09 (-0.27 to 0.08)	0.301	-0.05 (-0.31 to 0.19)	0.650	-0.12 (-0.28 to 0.04)	0.166
Corticosteroid therapy<br>- yes vs no	-0.02 (-0.19 to 0.16))	0.835	0.01 (-0.16 to 0.18)	0.903	-0.31 (-0.56 to -0.06)	**0.012**	-0.05 (-0.21 to 0.11)	0.542
Time from last active treatment<br>- ≤28 days vs >28 days	0.02 (-0.12 to 0.17)	0.713	-0.05 (-0.19 to 0.09)	0.500	0.01 (-0.20 to 0.20)	0.981	0.05 (-0.07 to 0.18)	0.417
Treatment type<br>- chemotherapy & ICI vs ICI	-0.07 (-0.26 to 0.10)	0.408	-0.09 (-0.28 to 0.08)	0.301	-0.10 (-0.36 to 0.15)	0.438	-0.18 (-0.35 to 0.01)	0.051

Statistically significant P values are highlighted in bold. *P* values derived from parametric 2-sided Wald's $\chi 2$ test with Bonferroni (α =0.01) correction for multiple comparisons. A two-sided *P* value of <0.05 was considered statistically significant. Log, logarithmic values; CI, confidence intervals; ECOG PS, Eastern Cooperative Oncology Group Performance Status; PD-L1 TPS, programmed cell death-ligand 1 tumor proportion score; ICI, immune checkpoint inhibitor. Corticosteroid therapy indicates ≥10 mg prednisone equivalent daily for at least 7 days in the 28 days preceding the third dose of vaccine; T helper cells, $CD3^{+}CD4^{+}$ cells; T cytotoxic cell, $CD3^{+}CD8^{+}$; B cells, $CD19^{+}$; NK, Natural killer, $CD56^{+}CD16^{+}$; Timepoint-1 denotes assessment before the third dose of tozinameran.

Supplementary Table S4. Multivariate analysis of peripheral lymphocyte counts by predefined clinical variables at timepoint-2

Covariate	T helper cell count (log)		T cytotoxic cell count (log)		B cell count (log)		NK cell count (log)	
	Beta (95% CI)	P value	Beta (95% CI)	P value	Beta (95% CI)	P value	Beta (95% CI)	P value
Sex<br>- male vs female	-0.02 (-0.20 to 0.15)	0.779	-0.08 (-0.27 to 0.09)	0.354	-0.01 (-0.22 to 0.19)	0.899	-0.03 (-0.24 to 0.17)	0.775
Age (years)<br>- >70 vs ≤70	0.12 (-0.04 to 0.28)	0.141	0.05 (-0.11 to 0.22)	0.528	-0.12 (-0.31 to 0.06)	0.204	0.03 (-0.15 to 0.23)	0.691
Cancer type<br>- lung vs others	-0.01 (-0.17 to 0.17)	0.994	-0.02 (-0.20 to 0.15)	0.776	-0.05 (-0.26 to 0.14)	0.574	-0.05 (-0.25 to 0.14)	0.609
ECOG PS<br>- 1 vs 0	0.03 (-0.14 to 0.21)	0.720	0.12 (-0.06 to 0.30)	0.195	-0.09 (-0.30 to 0.11)	0.382	0.07 (-0.13 to 0.28)	0.497
Number of metastatic sites<br>- ≥2 vs 1	0.06 (-0.11 to 0.24)	0.475	0.09 (-0.09 to 0.28)	0.307	0.02 (-0.19 to 0.23)	0.866	-0.01 (-0.23 to 0.19)	0.876
Brain metastases<br>- yes vs no	-0.01 (-0.17 to 0.17)	0.983	0.01 (-0.17 to 0.18)	0.937	0.11 (-0.09 to 0.31)	0.284	-0.10 (-0.30 to 0.10)	0.327
Bone metastases<br>- yes vs no	-0.04 (-0.22 to 0.13)	0.619	-0.07 (-0.25 to 0.11)	0.429	0.01 (-0.19 to 0.22)	0.900	-0.14 (-0.35 to 0.06)	0.166
Liver metastases<br>- yes vs no	-0.04 (-0.29 to 0.20)	0.711	0.15 (-0.10 to 0.40)	0.242	0.34 (0.05 to 0.64)	**0.019**	-0.15 (-0.44 to 0.13)	0.292
PD-L1 TPS<br>- >1% vs <1% or unknown	0.05 (-0.11 to 0.22)	0.535	-0.01 (-0.19 to 0.16)	0.905	-0.13 (-0.34 to 0.65)	0.183	0.10 (-0.09 to 0.31)	0.294
Metastatic treatment setting								

- first line vs second or later line	-0.29 (-0.48 to -0.09)	**0.003**	-0.38 (-0.58 to -0.18)	**0.001**	-0.24 (-0.47 to -0.01)	**0.040**	-0.20 (-0.43 to 0.02)	0.076
Corticosteroid therapy<br>- yes vs no	-0.06 (-0.25 to 0.12)	0.491	0.08 (-0.11 to 0.27)	0.407	-0.33 (-0.56 to -0.11)	**0.003**	0.01 (-0.21 to 0.22)	0.961
Time from last active treatment<br>- ≤28 days vs >28 days	0.01 (-0.13 to 0.17)	0.817	-0.02 (-0.18 to 0.14)	0.812	0.02 (-0.16 to 0.20)	0.800	0.09 (-0.08 to 0.28)	0.297
Treatment type<br>- chemotherapy & ICI vs ICI	-0.21 (-0.41 to -0.02)	**0.031**	-0.28 (-0.49 to -0.07)	**0.007**	-0.25 (-0.48 to -0.01)	**0.035**	-0.18 (-0.41 to 0.05)	0.128

Statistically significant P values are highlighted in bold. *P* values derived from parametric 2-sided Wald's $\chi 2$ test with Bonferroni (α =0.01) correction for multiple comparisons. A two-sided *P* value of <0.05 was considered statistically significant. Log, logarithmic values; CI, confidence intervals; ECOG PS, Eastern Cooperative Oncology Group Performance Status; PD-L1 TPS, programmed cell death-ligand 1 tumor proportion score; ICI, immune checkpoint inhibitor. Corticosteroid therapy indicates ≥10 mg prednisone equivalent daily for at least 7 days in the 28 days preceding the third dose of vaccine; T helper cells, $CD3^+CD4^+$ cells; T cytotoxic cell, $CD3^+CD8^+$; B cells, $CD19^+$; NK, Natural killer, $CD56^+CD16^+$; Timepoint-2 denotes assessment 28 days after the third dose of tozinameran.

Supplementary Figure S1. Flow cytometry analysis

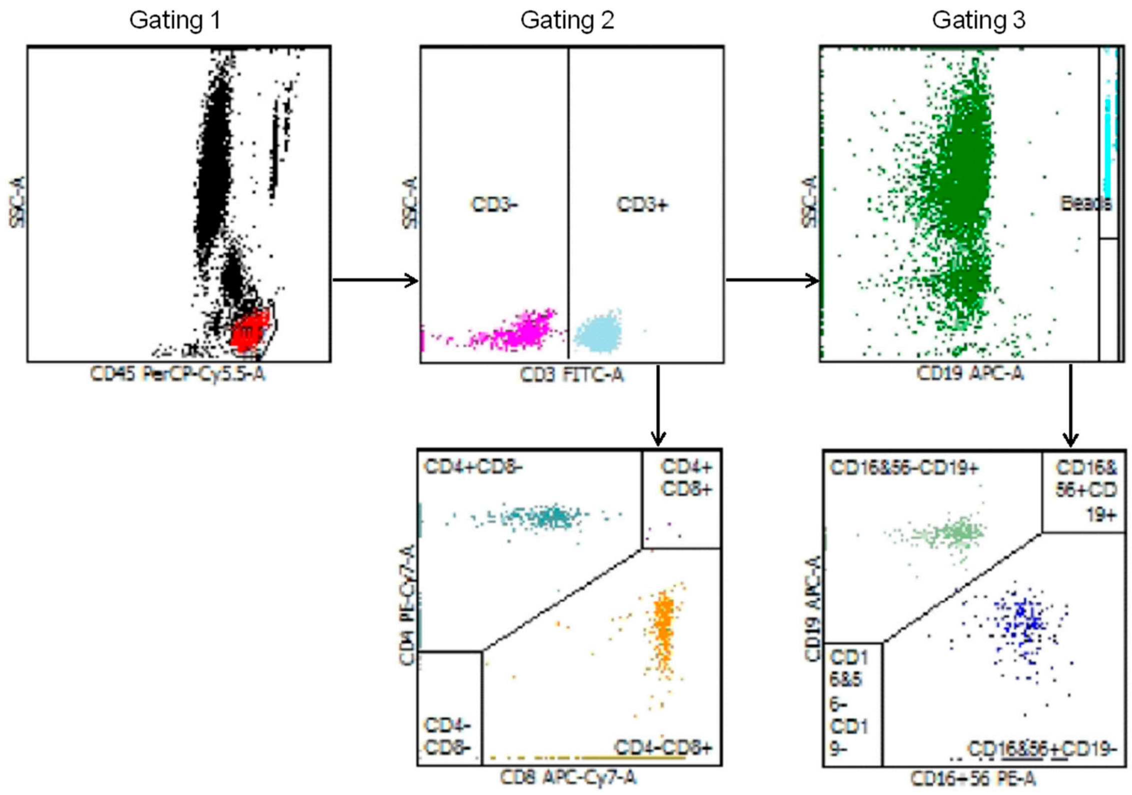

Operating procedure

Whole blood draws for flow cytometry analysis (3 mL/subject) were collected in ethylenediaminetetraacetic acid (EDTA) tubes. The BD Multitest 6-color TBNK reagent was used to determine absolute counts of B and NK, as well as CD4 and CD8 subpopulations of T cells. The panel for staining included the following monoclonal antibodies: CD3 FITC, CD4 PE-Cy7, CD8 APC-Cy7, CD19 APC, CD45 PerCP-Cy5.5, and CD56 PE + CD16 PE; BD Biosciences, San Jose, CA). The BD Trucount tubes (BD Biosciences, San Jose, CA) were filled with 20 μL of BD Multitest 6-color TBNK reagent and 50 μL aliquots of EDTA-anticoagulated whole blood. The mixture was incubated at room temperature in the dark for 20 minutes before being lysed with 2 mL of FACS Lysis Solution (BD Biosciences, San Jose, CA). After additional 15 minutes of incubation, the erythrocyte-lysed, unwashed, and stained samples were analyzed. The data were acquired using the BD FACSCanto II system and BD FACSCanto clinical software (BD Biosciences, San Jose, CA). The calibration of the instrument with BD FACS 7-color setup beads was confirmed before each

running process according to the manufacturer's instructions [1]. The results for each lymphocyte subset were reported as absolute cell counts/μL.

Gating strategy

Gating 1: first gating on a plot of forward scatter ($CD45^+$) and side scatter (SSC-A) to detect the absolute value of lymphocytes; Gating 2: second gating on plot a forward scatter ($CD3^+$) and side scatter (SSC-A) to detect the absolute value of T lymphocytes and then gated on the $CD8^+$ and $CD4^+$ to detect the absolute values of T helper cells ($CD3^+CD4^+$) and T cytotoxic cells ($CD3^+CD8^+$); Gating 3: third gating on a plot of forward scatter (CD19+) and side scatter (SSC-A) to detect the absolute value of B lymphocytes ($CD19^+$) and then gated on the $CD56^+CD16^+$ and CD19+ to detect the absolute values of NK cells ($CD56^+CD16^+$).

Supplementary Figure S2. Dynamic changes in absolute counts of peripheral lymphocyte subpopulations by PD-L1 TPS.

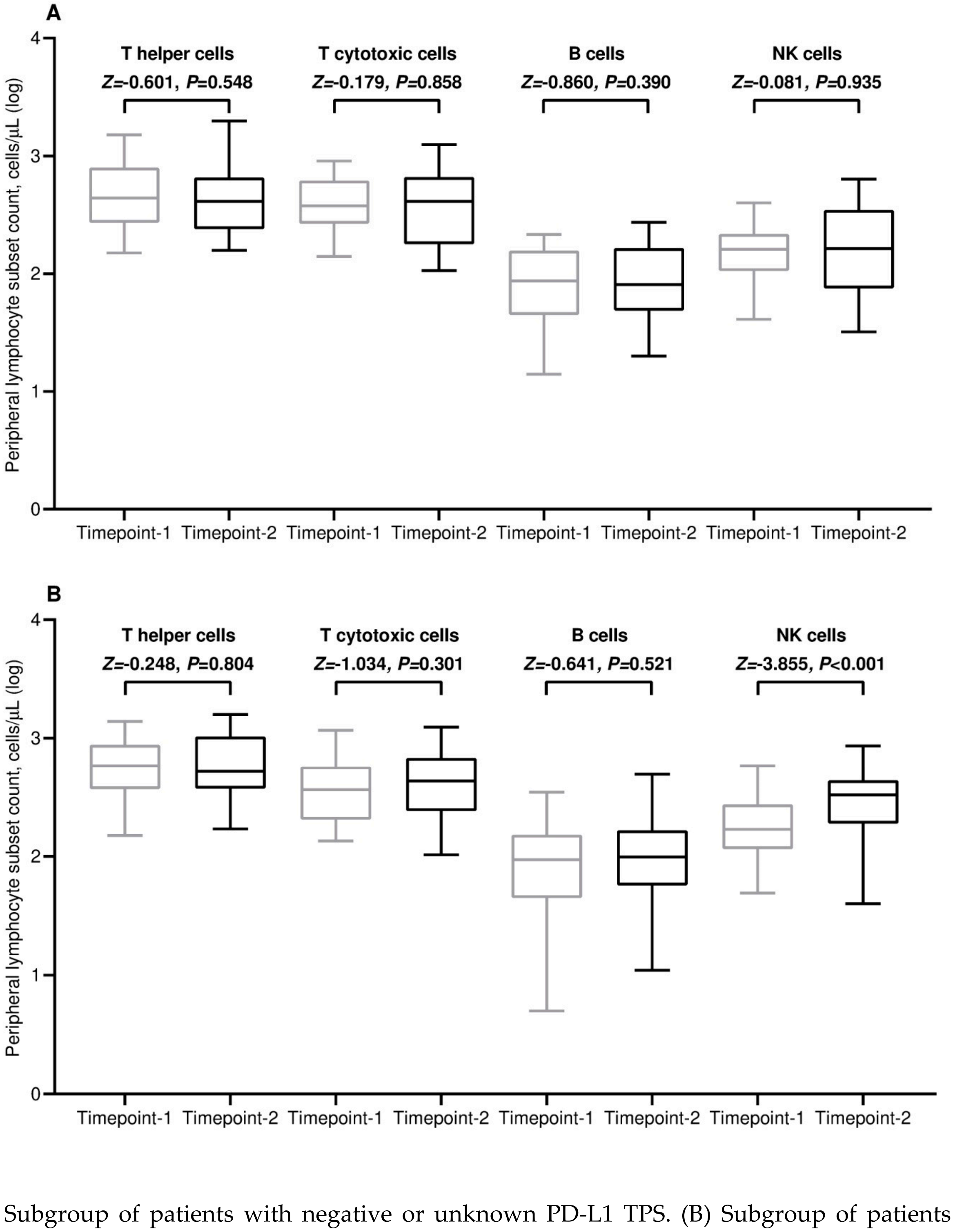

(A) Subgroup of patients with negative or unknown PD-L1 TPS. (B) Subgroup of patients with positive PD-L1 TPS. Bars denote median values with 95% confidence intervals. Differences between groups were assessed using the Wilcoxon signed-rank test. A two-sided P value <0.05 was considered statistically significant. PD-L1 TPS, programmed cell death-ligand 1 tumor proportion score; Log, logarithmic; T helper cells, $CD3^+CD4^+$ cells; T cytotoxic cell, $CD3^+CD8^+$; B cells, $CD19^+$;

NK, Natural killer, $CD56^{+}CD16^{+}$. Timepoint-1 indicates assessment before the third dose of tozinameran; timepoint-2 indicates assessment four weeks after the third dose of tozinameran.

Supplementary Figure S3. Longitudinal comparison of scatter plot distributions and medians of antibody titers

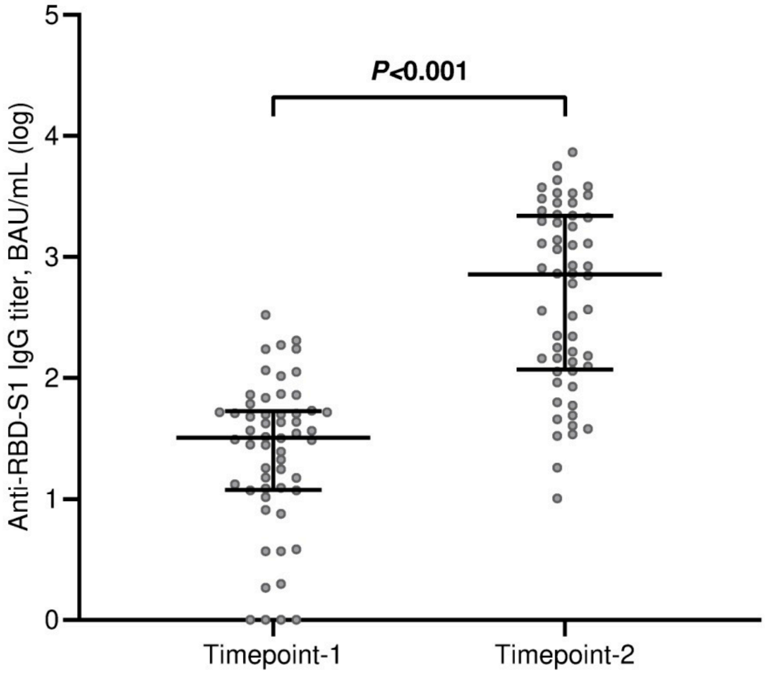

RBD-S1, receptor-binding domain (RBD) of the SARS-CoV-2 Spike protein (S1); BAU, Binding Antibody Unit; log, logarithmic values. Bars indicate median values with interquartile range. Timepoint-1 denotes assessment before the third dose of tozinameran; timepoint-2 denotes assessment four weeks after the third dose of tozinameran.

Supplementary Figure S4. Correlation between antibody titers and lymphocyte subpopulation counts before the third dose of tozinameran

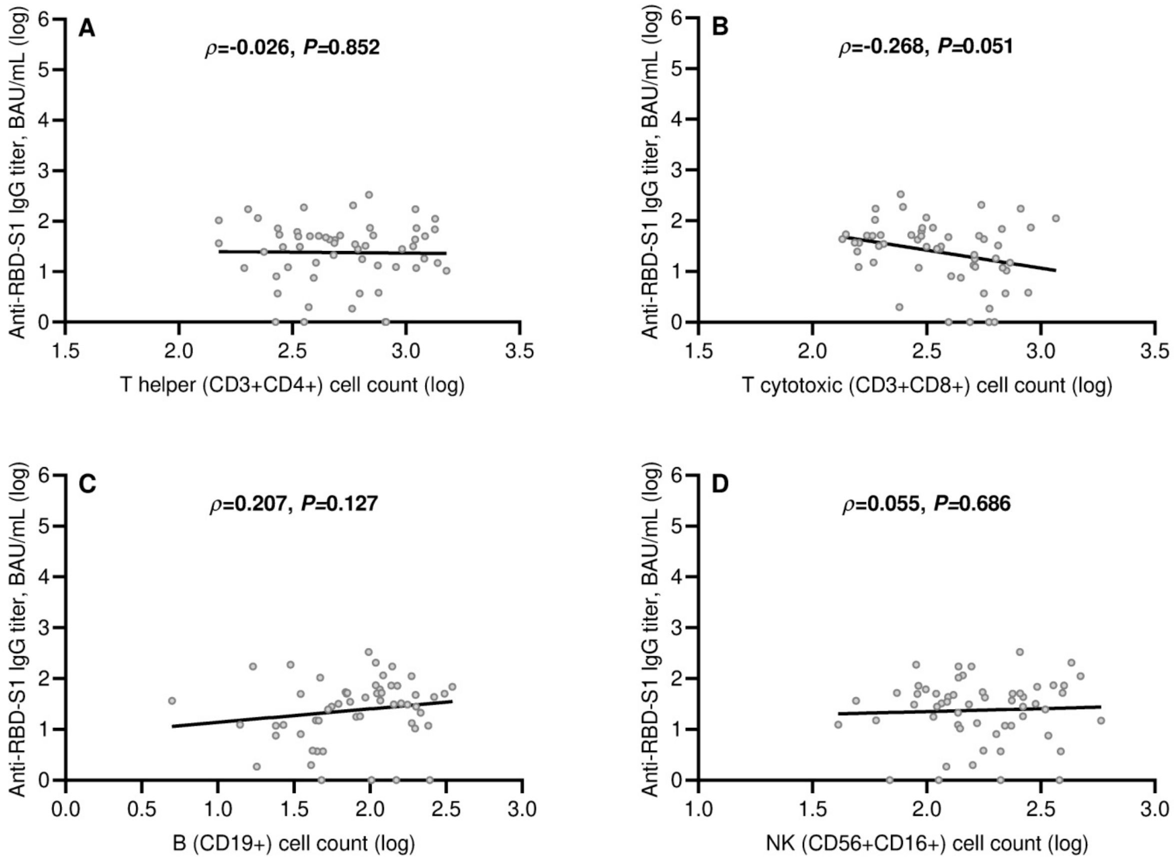

(A) T helper cells ($CD3^{+}CD4^{+}$): ϱ=-0.026 (95% CI -0.312 to 0.247), p=0.852. (B) T cytotoxic cells ($CD3^{+}CD8^{+}$); ϱ=-0.268 (95% CI -0.521 to 0.004) p=0.051. (C) B cell ($CD19^{+}$): ϱ=0.207 (95% CI -0.084 to 0.478) p=0.127. (D) NK cells ($CD56^{+}CD16^{+}$): ϱ=0.055 (95% CI -0.215 to 0.326), p=0.686. Correlation was assessed with the Spearman's test; a two-sided P value <0.05 was considered statistically significant. RBD-S1, receptor-binding domain (RBD) of the SARS-CoV-2 Spike protein (S1); BAU, binding antibody unit; log, logarithmic values; CI, confidence interval.

Supplementary Figure S5. Correlation between antibody titers and lymphocyte subpopulation counts after the third dose of tozinameran

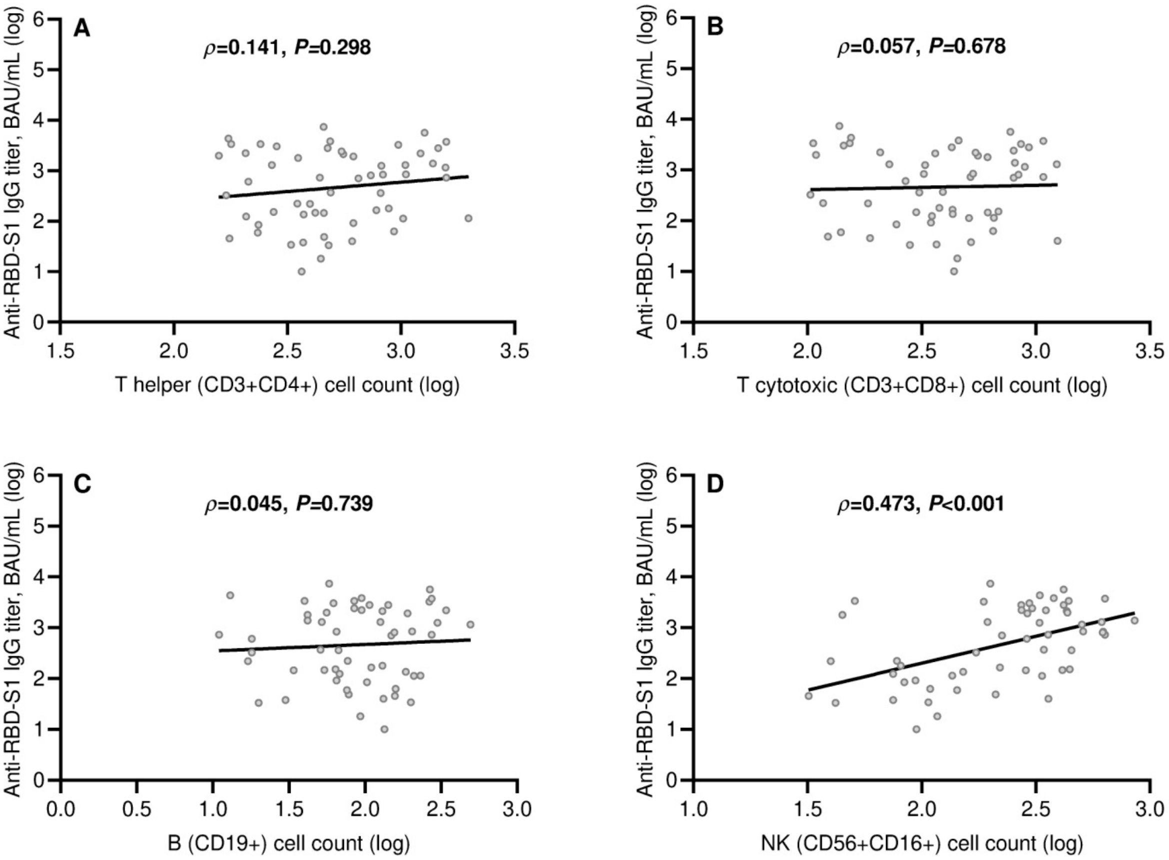

(A) T helper cells ($CD3^+CD4^+$): $\varrho=0.141$ (95% CI -0.116 to 0.402), p=0.298. (B) T cytotoxic cells ($CD3^+CD8^+$): $\varrho=0.057$ (95% CI -0.232 to 0.336), p=0.678 . (C) B cell ($CD19^+$): $\varrho=0.045$ (95% CI -0.202 to 0.306), p=0.739. (D) NK cells ($CD56^+CD16^+$): $\varrho=0.473$ (95% CI 0.220 to 0.655), p<0.001. Correlation was assessed with the Spearman's test; a two-sided P value <0.05 was considered statistically significant. RBD-S1, receptor-binding domain (RBD) of the SARS-CoV-2 Spike protein (S1); BAU, binding antibody unit; log, logarithmic values; CI, confidence interval.

Supplementary Figure 6. Time-to-event depending on clinical benefit outcome

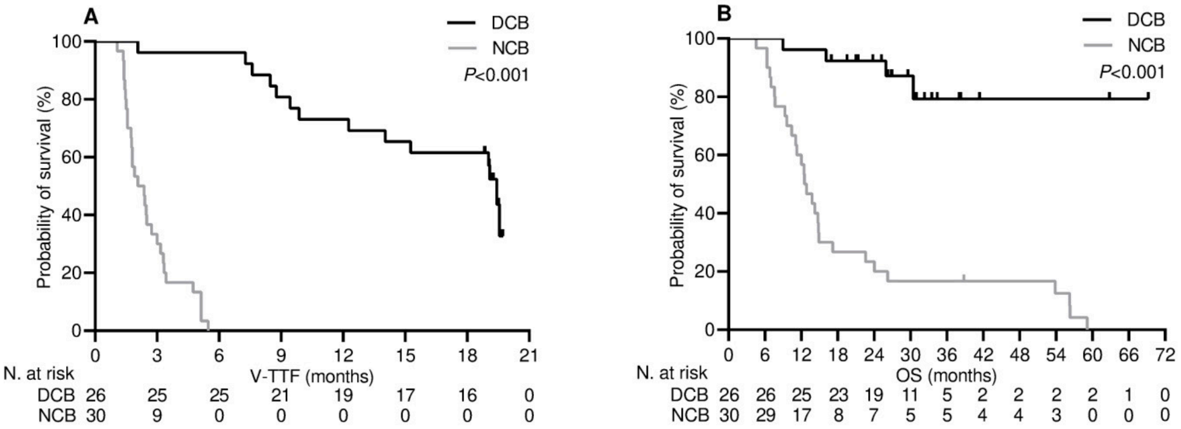

(A) Vaccine-related time-to-treatment failure and (B) overall survival: DCB (durable clinical benefit) vs NCB (no clinical benefit

References

1. BD FACSCanto™ Software. https://www.bdbiosciences.com/en-eu/products/software/instrument-software/bd-facscanto-clinical-software/; [Accessed 4 June 2023].
